# Cell Model of Catecholaminergic Polymorphic Ventricular Tachycardia Reveals Early and Delayed Afterdepolarizations

**DOI:** 10.1371/journal.pone.0044660

**Published:** 2012-09-04

**Authors:** Kirsi Kujala, Jere Paavola, Anna Lahti, Kim Larsson, Mari Pekkanen-Mattila, Matti Viitasalo, Annukka M. Lahtinen, Lauri Toivonen, Kimmo Kontula, Heikki Swan, Mika Laine, Olli Silvennoinen, Katriina Aalto-Setälä

**Affiliations:** 1 Institute of Biomedical Technology, University of Tampere, Tampere, Finland; 2 BioMediTech, Tampere, Finland; 3 Minerva Foundation Institute for Medical Research, Helsinki, Finland; 4 Department of Cardiology, Helsinki University Hospital, Helsinki, Finland; 5 Research Program’s Unit Molecular Medicine, University of Helsinki, Helsinki, Finland; 6 Department of Medicine, University of Helsinki, Helsinki, Finland; 7 Tampere University Hospital, Tampere, Finland; 8 Heart Center, Tampere, Finland; Georgia State University, United States of America

## Abstract

**Background:**

Induced pluripotent stem cells (iPSC) provide means to study the pathophysiology of genetic disorders. Catecholaminergic polymorphic ventricular tachycardia (CPVT) is a malignant inherited ion channel disorder predominantly caused by mutations in the cardiac ryanodine receptor (RyR2). In this study the cellular characteristics of CPVT are investigated and whether the electrophysiological features of this mutation can be mimicked using iPSC -derived cardiomyocytes (CM).

**Methodology/Principal Findings:**

Spontaneously beating CMs were differentiated from iPSCs derived from a CPVT patient carrying a *P2328S* mutation in RyR2 and from two healthy controls. Calcium (Ca^2+^) cycling and electrophysiological properties were studied by Ca^2+^ imaging and patch-clamp techniques. Monophasic action potential (MAP) recordings and 24h-ECGs of *CPVT-P2328S* patients were analyzed for the presence of afterdepolarizations. We found defects in Ca^2+^ cycling and electrophysiology in CPVT CMs, reflecting the cardiac phenotype observed in the patients. Catecholaminergic stress led to abnormal Ca^2+^ signaling and induced arrhythmias in CPVT CMs. CPVT CMs also displayed reduced sarcoplasmic reticulum (SR) Ca^2+^ content, indicating leakage of Ca^2+^ from the SR. Patch-clamp recordings of CPVT CMs revealed both delayed afterdepolarizations (DADs) during spontaneous beating and in response to adrenaline and also early afterdepolarizations (EADs) during spontaneous beating, recapitulating the changes seen in MAP and 24h-ECG recordings of patients carrying the same mutation.

**Conclusions/Significance:**

This cell model shows aberrant Ca^2+^ cycling characteristic of CPVT and in addition to DADs it displays EADs. This cell model for CPVT provides a platform to study basic pathology, to screen drugs, and to optimize drug therapy.

## Introduction

Catecholaminergic polymorphic ventricular tachycardia (CPVT) is a severe inherited cardiac disorder characterized by stress-induced polymorphic ventricular tachycardia in a structurally normal heart. Approximately 30% of CPVT patients have symptoms before the age of 10 and the mortality rate is 30–35% by the age of 30. β-blockers are recommended for CPVT, but this treatment often fails to prevent even fatal arrhythmias [1].

CPVT is caused by mutations in the cardiac ryanodine receptor (*RyR2*) or calsequestrin (*CASQ2*) gene. RyR2 is involved in the release of calcium (Ca^2+^) from the sarcoplasmic reticulum (SR) and thus plays a key role in excitation-contraction coupling. Calsequestrin is a regulatory calcium-buffering protein associated with RyR2 in the SR. *RyR2* mutations can be detected in about 70% of patients with CPVT. These mutations are thought to result in increased release, or leak, of Ca^2+^ from the SR potentially leading to diastolic oscillations of intracellular Ca^2+^, delayed afterdepolarizations (DAD), and polymorphic ventricular tachycardia [1]. However, our understanding of the detailed pathophysiology behind CPVT remains incomplete.

Although the pathomechanisms have been clinically studied in CPVT patients with exercise stress tests, genetically engineered mouse models have been significant to the understanding of CPVT. Most of the CPVT-studies related to *RyR2* mutations have been performed in autosomal dominant transgenic knock-in mouse models expressing mutations which have shown Ca^2+^ -mediated arrhythmogenesis [2].

Induced pluripotent stem cell (iPSC) technology where pluripotent stem cells are generated by reprogramming differentiated cells into a pluripotent state provides a way to study the pathophysiology of various disorders in human cells. iPSCs can be differentiated into the desired cell type, retaining the original genotype. Recently CPVT-specific iPSCs -derived cardiomyocytes (CMs) from individuals carrying *RyR2* mutations [3], [4] have demonstrated DADs as the electrical abnormalities.

The *P2328S* mutation in *RyR2* has been found in families with CPVT. Here we introduce a functional cell model for CPVT caused by this mutation. We investigated the mechanistic characteristics of this disease *in vitro* using iPSC –derived CMs. Importantly, we demonstrate the presence of EADs in addition to DADs as a pathophysiological mechanism of CPVT.

## Methods

### Generation of Patient-Specific iPSCs

The study was approved by the ethical committee of Pirkanmaa Hospital District (R08070) and written informed consent was obtained from all the participants. Patient-specific iPSC lines were established as described earlier [5]. Two CPVT-specific iPSC lines (UTA.05203.CPVT and UTA.05208.CPVT) were generated from a 25-year-old male carrying a *RyR2-P2328S* mutation. iPSC lines UTA.00112.hFF (derived from foreskin fibroblasts) and UTA.04602.WT (from skin fibroblasts of a healthy 55-year-old female) were used as controls.

**Table 1:** Primer sequences for RT-PCR.

Gene	Forward Primer	Reverse Primer	GenBank ID
**Endodermal**			
*AFP*	AGAACCTGTCACAAGCTGTG	GACAGCAAGCTGAGGATGTC	174
**Ectodermal**			
*Nestin*	CAGCTGGCGCACCTCAAGATG	AGGGAAGTTGGGCTCAGGACTGG	10763
**Mesodermal**			
*a-cardiactin*	GGAGTTATGGTGGGTATGGGTC	AGTGGTGACAAAGGAGTAGCCA	70
**Ca^2+^ cycling**			
*RyR2*	TAGATTTATAAGGGGCCTTG	GATTCTTCAGGGCTCGTAGT	6262
*Cav1.2*	TGACATCGAGGGAGAAAACT	ACATTAGACTTGACTGCGGC	775
*Serca2a*	GAGAACGCGCACACCAAGA	TTGGAGCCCCATCTCTCCTT	488
*Phospholamban*	CTGCCAAGGCTACCTAAAAG	AGCTGAGCGAGTGAGGTATT	5350
*NCX*	TTCCAGAATGATGAAATTGTGAAGAT	TCCTCAAGCACAAGGGAGAAAC	6546
*TNTT2*	ATCCCCGATGGAGAGAGAGT	TCTTCTTCTTTTCCCGCTCA	7139
*GAPDH*	AGCCACATCGCTCAGACACC	GTACTCAGCGGCCAGCATCG	2597

### Characterization of iPSC Lines

#### Genotyping

The *RyR2-P2328S* mutation was assayed with PCR amplification of genomic DNA with primers for *RyR2* exon 46 (forward: ttt gtt tac tta tct tcc cca ttc, reverse: tat gga tca ctc gtg agg gt) and HaeIII digestion (New England Biolabs, Ipswich, MA, USA). DNA for wild type was 170 and 87 and for *P2328S* heterozygote 257, 170 and 87 base pairs long. For confirmation of the mutation by direct sequencing, the *RyR2* exon 46 PCR products were sequenced with BigDye Terminator v3.1 and ABI 3730xl DNA Analyzer (Applied Biosystems, Carlsbad, CA, USA).

**Figure 1:**
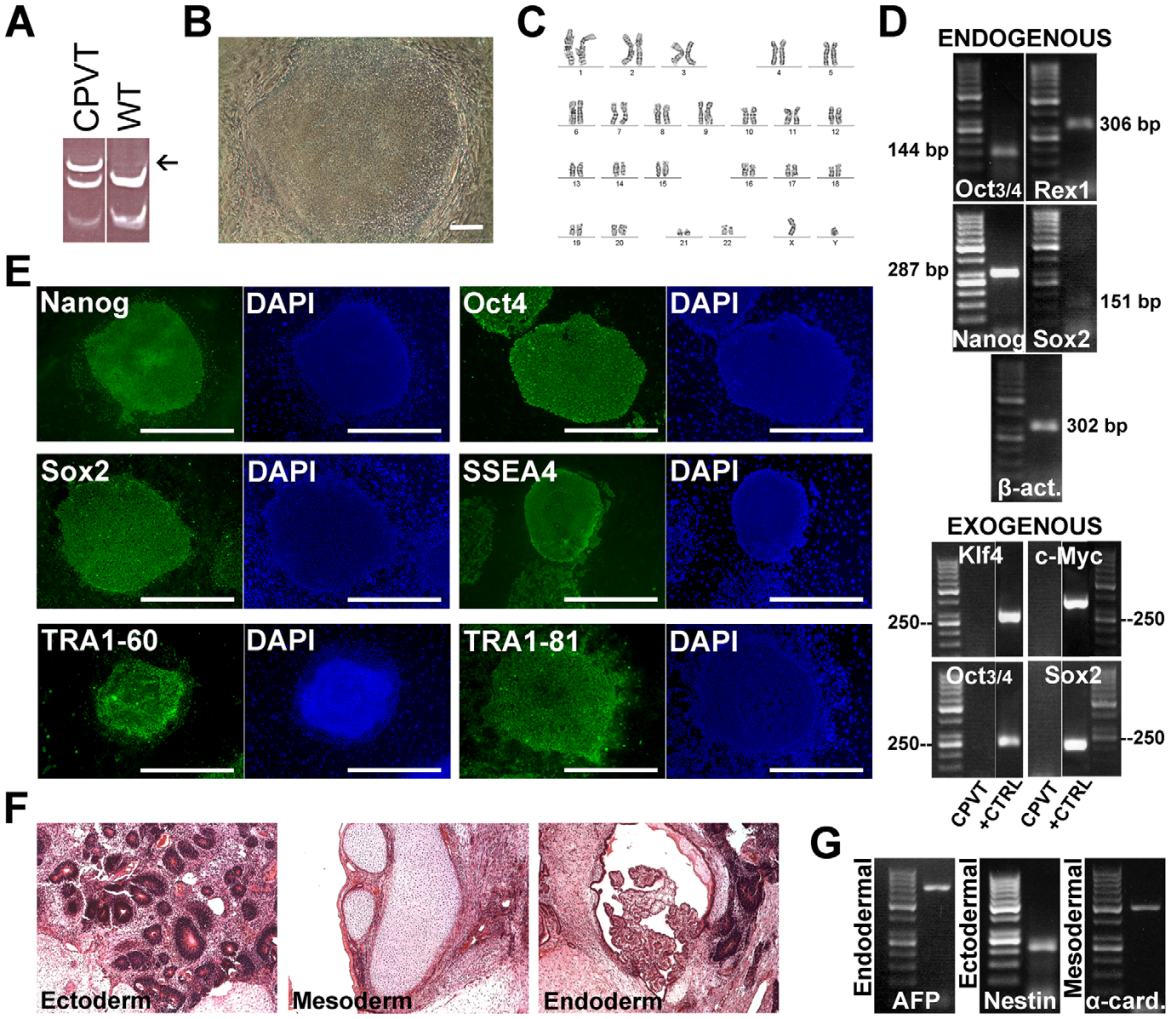
Characterization of CPVT-iPSCs. **A**, Mutation analysis confirming the RyR2-*P2328S* mutation with altered DNA cleavage (arrow). **B**, Morphology of an iPSC colony. Scale bar 200 µm. **C**, Normal karyotype. **D**, Expression of pluripotency markers at passage 4 shown by RT-PCR, β-actin serving as a housekeeping gene. All studied endogenous pluripotency genes are turned on. None of the exogenous genes are expressed at passage 4. **E**, Immunocytochemical staining showing expression of pluripotency markers. Scale bars 1000 µm. **F**, Teratomas made from a CPVT-iPSC line further confirms pluripotency. **G**, EBs express markers from all the three embryonic germ layers.

#### Karyotype analysis

Karyotypes of the cell lines were determined using standard G-banding chromosome analysis (Medix laboratories, Espoo, Finland).

#### Reverse transcription polymerase chain reaction (RT-PCR)

Endogenous and exogenous gene expressions were studied from iPSCs by RT-PCR. The PCR reaction consisted of 1 µl cDNA and 500 nmol/L of each primer. PCR primers for iPSC characterization and detailed reaction conditions have been described earlier [5]. β-actin served as a housekeeping gene.

**Figure 2:**
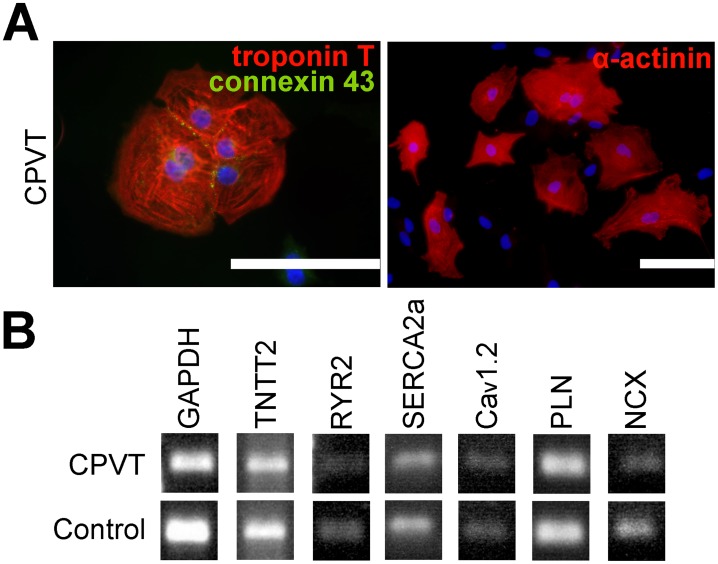
Characterization of iPSC-derived CPVT CMs. **A**, Immunocytochemical staining of cardiac markers, blue represents DAPI-staining for nuclei. Scale bars 100 µm. **B**, The expression of Ca^2+^ cycling genes in differentiated CMs shown by RT-PCR. GAPDH is used as a housekeeping gene.

#### Immunocytochemistry

The iPSCs were fixed with 4% paraformaldehyde (Sigma-Aldrich, Saint Louis, USA). Primary antibodies anti-SOX2, anti-NANOG, anti-stage-specific embryonic antigen (SSEA)4, and anti-tumour-related antigen (TRA)1–81 (all 1∶200, from Santa Cruz Biotechnology, Santa Cruz, CA, USA), anti-OCT3/4 (1∶400, R&D Systems) and anti-TRA1–60 (1∶200, Millipore) were used. Cells were mounted with Vectashield (Vector Laboratories, USA) containing 4′, 6-diamidino-2-phenylindole (DAPI) for staining nuclei.

**Figure 3:**
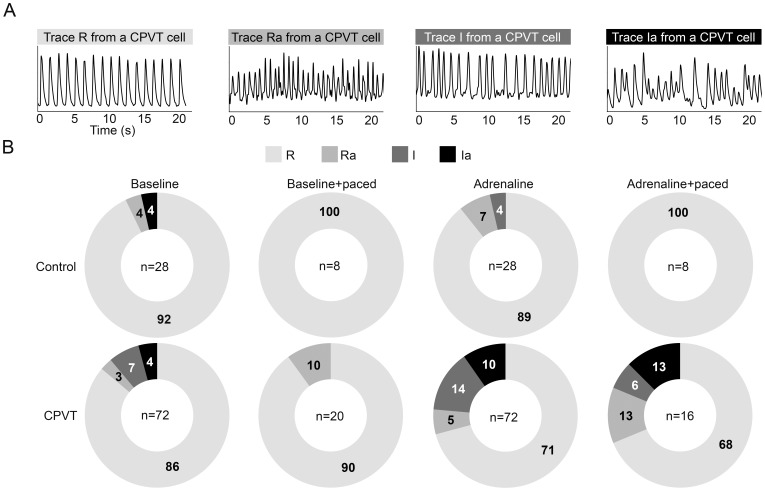
Intracellular Ca^2+^ cycling and analysis of rhythm. **A**, representative traces of the four different rhythm categories. Regular rhythm of calcium release with stable amplitude R, regular rhythm with varying amplitude Ra, irregular rhythm with stable amplitude I, irregular rhythm with varying amplitude Ia. **B**, doughnut charts indicating the percentage of CPVT and control CMs under each rhythm category.

#### Embryoid body (EB) formation

EBs were maintained in EB-medium (KO-DMEM with 20% FBS, NEAA, L-glutamine and penicillin/streptomycin) for 5 weeks. The expression of markers characteristic of ectoderm (*Nestin*), endoderm (*AFP*), and mesoderm (*α-cardiactin*) development in EBs were studied (primers in Table 1).

**Figure 4:**
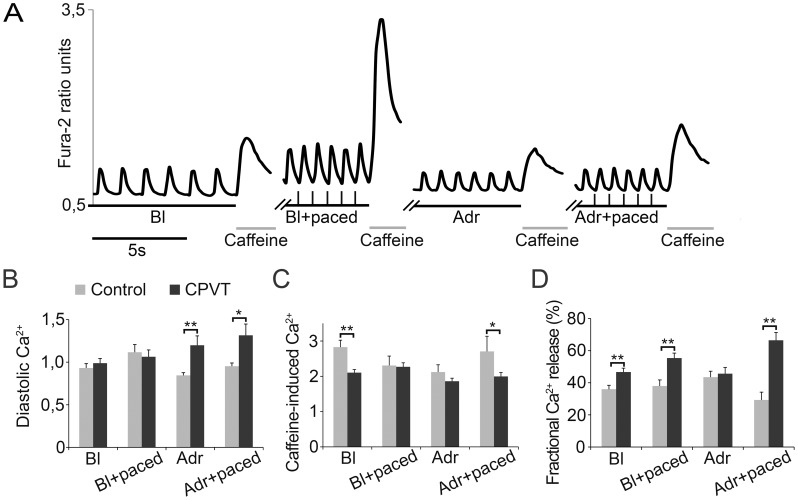
Intracellular Ca^2+^ cycling and SR Ca^2+^ stores. **A**, representative traces from a single CPVT cell demonstrating the experimental protocol. Bl; spontaneous baseline beating, Adr; adrenaline perfusion. Caffeine was added following 10 spontaneous or paced beats. **B**, diastolic level of intracellular Ca^2+^. **C**, amplitude of caffeine-induced Ca^2+^ transients. **D**, amplitude of Ca^2+^ transients divided by amplitude of caffeine-induced Ca^2+^ transient, indicating fractional SR Ca^2+^ release. Units in A and B are Fura-2 ratio units, in C ΔF/F0. Numbers of control vs CPVT CMs analyzed: Bl n = 54 vs n = 90, Bl+paced n = 25 vs n = 50, Adr n = 27 vs 47, Adr+paced n = 19 vs n = 35, respectively. Error bars, SEM. *P<0.05, **P<0.01, with student's t-test.

#### Teratoma formation

The study was approved by ELLA- Animal Experiment Board of Regional State Administrative Agency for Southern Finland (ESAVI/6543/04.10.03/2011). iPSCs were injected into nude mice under the testis capsule and tumor samples collected 8 weeks after injection. This was followed by fixation with 4% paraformaldehyde and staining of the sections with haematoxylin and eosin.

**Figure 5:**
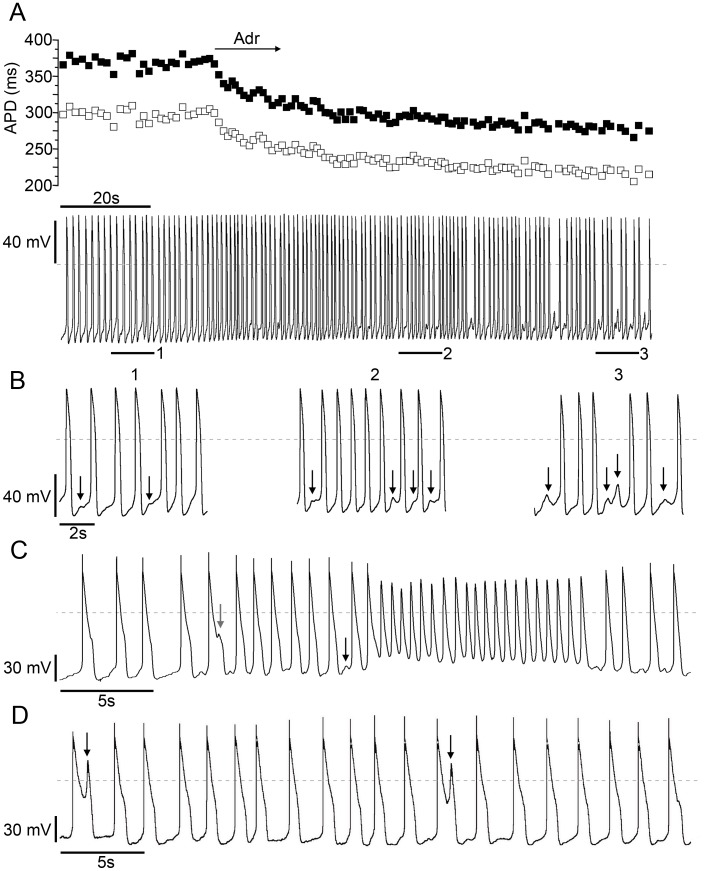
CPVT-*P2328S* CMs display DADs and EADs. A , Time course of APD_50_ (empty squares) and APD_90_ (filled squares) Adr indicates perfusion with adrenaline. **B**, bars 1–3 are 9 sec time courses enlarged from **A**. (1) baseline with DADs, (2) DADs in the presence of adrenaline, (3) DADs continue after adrenaline perfusion. MDP −70 mV. Arrows indicate DADs. **C**, A CPVT-*P2328S* CM showing an EAD (grey arrow) and a DAD (black arrow) followed by a spontaneous burst episode (MDP −50 mV, maximum upstroke amplitude 45 mV). **D**, Current clamp recording of a CPVT-*P2328S* CM showing occasional EADs (arrows). MDP −70 mV. Dashed lines indicate the zero reference potential.

**Table 2:** Characteristics of spontaneous ventricular-like APs in control and CVPT-CMs during regular beating.

Baseline	BPM	APD50 (ms)	APD90 (ms)	APA (mV)	MDP (mV)
control (n = 16)	41±6	204.4±20.3	329.7±22.4	117.85±2.61	−68.35±1.87
CPVT (n = 14)	43±5	238.6±22.4	305.4±25.7	114.80±2.70	−67.55±1.51
**Adrenaline**	**BPM** **% increase**	**APD50** **% decrease**	**APD90** **% decrease**	**APA** **ΔmV**	**MDP** **ΔmV**
control (n = 5)	23.2±0.9	18.4±1.8	16.8±2.1	−5.82±1.17	+2.55±0.61
CPVT (n = 11)	16.0±12.3	21.5±5.5	24.0±3.5	−8.25±2.15	+4.59±0.82

### Cardiomyocyte Differentiation and Characterization

Differentiation into cardiomyocytes (CMs) was carried out by co-culturing iPSCs with murine visceral endoderm-like (END-2) cells (Humbrecht Institute, Utrecht, The Netherlands) as described earlier [6]. The beating areas of the cell colonies were mechanically excised and treated with collagenase A (Roche Diagnostics) [6].

#### Immunocytochemistry

Single beating CMs were immunostained with anti-cardiac-troponin-T (1∶1500, Abcam, Cambridge, MA, USA), anti-α-actinin (1∶1500, Sigma-Aldrich) and anti-connexin-43 (1∶1000, Sigma-Aldrich).

**Figure 6:**
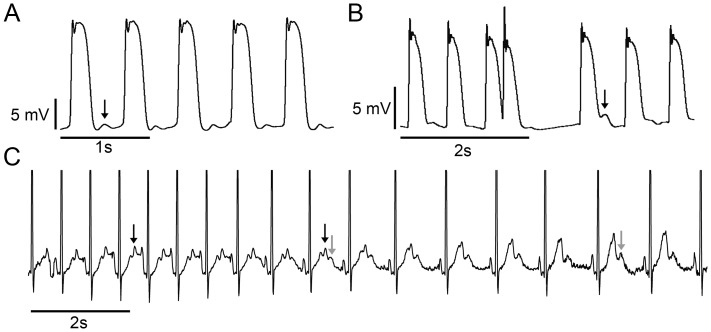
CPVT patients show afterdepolarizations and corresponding ECG changes. A , MAP recording from CPVT-*P2328S* patient showing DADs (arrow). **B**, Example MAP recording from a CPVT-*P2328S* patient showing an extrasystole and the following beat with EAD (arrow). **C**, Example 24-h ECG recording from a CPVT-P2328S patient showing the simultaneous occurrence of T2- (black arrow) and U-waves (grey arrow) representing clinical counterparts to EADs [11], [28] and DADs [8], respectively.

#### RT-PCR for cardiac Ca^2+^-cycling protein analysis

RNA was isolated from iPSC-derived CMs. The expression of troponin T (*TNTT2*), *RyR2*, SR Ca^2+^ ATPase (*SERCA2a*), L-type Ca^2+^ channel (*Ca_v_1.2*), phospholamban (*PLN*) and sodium-calcium exchanger (*NCX*) were assessed. Primer sequences are listed in Table 1. *GAPDH* served as a housekeeping gene.

#### Ca^2+^ imaging

Dissociated CMs on a coverslip were loaded with 4 µmol/L Fura-2 AM (Invitrogen, Molecular Probes) for 30 minutes in HEPES based medium, followed by a 30-minute de-esterification. The coverslip was then transferred to an RC-27NE or an RC-25 recording chamber (Warner Instruments Inc., CT, USA) and continuously perfused with 37°C HEPES based perfusate. The perfusate was preheated by an SH-27B inline-heater controlled by a TC-324B unit (Warner Instruments Inc., USA) and consisted of (in mmol/L): 137 NaCl, 5 KCl, 0.44 KH_2_PO_4_, 20 HEPES, 4.2 NaHCO_3_, 5 D-glucose, 2 CaCl_2_, 1.2 MgCl_2_ and 1 Na-puryvate (pH was adjusted to 7.4 with NaOH).

Ca^2+^ measurements were conducted on an inverted IX70 microscope (Olympus Corporation, Hamburg, Germany) where spontaneously beating CMs were visualized with a UApo/340 x20 air objective (Olympus). Images were acquired with an ANDOR iXon 885 CCD camera (Andor Technology, Belfast, Northern Ireland) synchronized with a Polychrome V light source by a real time DSP control unit and TILLvisION software (TILL Photonics, Munich, Germany). Fura-2 in CMs was excited at 340 nm and 380 nm light and the emission was recorded at 505 nm. For Ca^2+^ analysis, regions of interests were selected for spontaneously beating cells and background noise was subtracted before further processing. The Ca^2+^ levels are presented as ratiometric values of F340/F380 or as ΔF/F_0_.

CMs were paced with 10–50 ms pulses (27–32 mA) (DS3 Constant Current/Voltage Isolated Stimulators, Digitimer LTD, USA) at a frequency of 0.1–0.3 Hz higher than the spontaneous beating rate. The changes in Ca^2+^ were recorded during spontaneous baseline beating, electrical pacing, spontaneous beating during 1 µmol/L adrenaline (Sigma-Aldrich) perfusion and electrical pacing during adrenaline perfusion. The SR Ca^2+^ content was measured by releasing all the SR Ca^2+^ with instantaneous and high concentration (40 mmol/L) caffeine (Sigma-Aldrich) puffs after each measurement, after which the relative amplitude change in calcium release in CPVT versus control CMs was quantified. Viability of CMs was confirmed after the experimental protocol. Amplitudes, beating frequency and caffeine induced Ca^2+^ peaks were analyzed with Clampfit version 9.2 (Molecular devices, USA). Analysis was performed blinded to genotype of CMs.

#### Measurement of action potentials

Action potentials (APs) were recorded in current-clamp mode using the standard patch-clamp technique in the perforated patch configuration [7]. The HEPES based extracellular perfusate for current-clamp recordings consisted of (in mmol/L): 143 NaCl, 5 KCl, 1.8 CaCl_2_, 1.2 MgCl_2_, 5 glucose, 10 HEPES, pH was adjusted to 7.4 with NaOH and the osmolarity set to 300±2 mOsm (Gonotec, Osmomat 030, Labo Line Oy, Helsinki, Finland). The intracellular solution consisted of (in mmol/L): 122 KMeSO_4_, 30 KCl, 1 MgCl_2_, 10 HEPES. KOH was used to set pH to 7.15 and the osmolarity was set to 295±2 mOsm. Amphotericin B (Sigma-Aldrich) was used as membrane perforation agent and dissolved in DMSO to a final concentration in the patch pipette of 0.24 mg/ml. Spontaneously beating CMs were patched in same bath conditions as in Ca^2+^ imaging. Patch pipettes (model PG150T, Harvard Apparatus, UK) were pulled with a PC-10 puller and flame polished with Microforge MF-900 (Narishige, UK) to a resistance of 2.0–2.5 MΩ measured in the bath perfusate. APs were recorded in gab-free mode with pClamp 10.2 using the Axopatch 200B patch-clamp amplifier connected to an acquisition computer via AD/DA Digidata 1440 (Molecular devices, USA). Current-clamp recordings were digitally sampled at 20 kHz and filtered at 5 kHz using the lowpass Bessel filter on the recording amplifier. AP duration at 50% and 90% of repolarization (ADP_50_ and ADP_90_), AP amplitude (APA), maximum diastolic potential (MDP) and beats per minute (BPM) were extracted from AP recordings using an automated script in Microcal OriginTM 8.6.

### Monophasic Action Potential Recordings

Monophasic action potentials (MAPs) were previously recorded from CPVT patients and healthy controls as reported [8]. Briefly, MAPs were recorded from the right ventricular septum with a bipolar silver-silver chloride catheter (model 006248, Bard Inc., Lowell, MA, USA). Data were recorded during sinus rhythm and atrial pacing at a constant cycle length of 600 ms, both during baseline and adrenaline infusion (maximum rate 0.05 µg/kg/min). Custom-made software was used for analysis.

### Definition of DADs and EADs

EADs were defined as low-amplitude depolarizations that occur during phase 2 or 3 of the AP, before completion of repolarization, and have an amplitude of ≥3% of the preceding AP. DADs were defined as low-amplitude depolarizations that occur after completion of repolarization, and have an amplitude of ≥3% of the preceding AP [9].

### 24h-ECG Recordings

24h-ECGs were previously recorded from CPVT patients and healthy controls as reported [10]. Briefly, 24-h ECGs were recorded using commercial tape recorders (model 8500; Marquette Electronics Inc., Milwaukee, WI, USA). The tapes were initially analyzed with a Marquette 8000 Holter Analysis system (version 5.8 software) to label the QRS complexes to normal, ventricular extrasystoles, or aberrant complexes.

### Definition of T1-, T2-, and U-waves

The first peak during repolarization was considered as a T1-wave. The second peak was considered as a T2-wave if it occasionally merged with the T1-wave, or as a U-wave if it never merged with the T1-wave. The third peak, which never merged with the T1-wave, was also considered as a U-wave [10], [11], [12].

### Statistical Analysis

The significance of differences between two groups was evaluated with the unpaired Student’s *t*-test. The significance of changes within a group was evaluated with the paired Student’s *t*-test. *P*<0.05 was considered statistically significant, where (*) represents *P*<0.05 and (**) *P*<0.01. Data are expressed as means ± S.E.M. and n (where indicated) refers to the number of cells or experiments.

## Results

### Characterization of iPSC Lines Confirms Pluripotent Stem Cell Characteristics

The presence of the *P2328S* mutation was confirmed in the two *CPVT-P2328S* iPSC lines (Figure 1A). The iPSC colonies were morphologically round-shaped and the iPSC lines had normal karyotype (Figure 1B–C). All studied endogenous pluripotency genes were turned on and expression of retrovirally encoded reprogramming factors was silenced (Figure 1D). iPSC lines expressed endogenous pluripotent markers at the protein level (Figure 1E). Pluripotency was confirmed by teratoma formation and with *in vitro* embryoid body (EB) formation expressing all three germ layers (Figure 1F–G).

### iPSC-derived CMs Express Cardiac Markers

iPSCs were differentiated into spontaneously beating cells and the differentiated CMs expressed cardiac markers at the protein level (Figure 2A). RT-PCR was performed to confirm the expression of genes related to Ca^2+^ cycling (Figure 2B).

### *CPVT-P2328S* CMs Display Aberrant Ca^2+^ Cycling

Ca^2+^ cycling properties of CPVT and control CMs were compared in four conditions: spontaneous baseline beating, pacing, spontaneous beating during adrenaline perfusion, and pacing during adrenaline perfusion. Ca^2+^ cycling was categorized into four different rhythm categories in which three of them the Ca^2+^ cycling was characterized abnormal due to varying amplitude and/or irregular rhythm (Figure 3A). Ca^2+^ cycling abnormalities were more common in CPVT CMs than in control CMs in each studied condition (Figure 3B). At baseline a higher percentage of CPVT CMs (14%) showed abnormal Ca^2+^ cycling when compared to control CMs (8%). Pacing stabilized Ca^2+^ cycling partially in CPVT CMs and completely in control CMs. Adrenaline increased Ca^2+^ cycling abnormalities to 30% of the CPVT CMs. In control CMs adrenaline had no effect on Ca^2+^ cycling. Pacing with adrenaline abolished all Ca^2+^ cycling abnormalities in controls but did not have an effect in CPVT CMs.

Under baseline and electrical pacing conditions, CPVT and control CMs presented similar diastolic Ca^2+^ levels (Figure 4B). However, adrenaline with and without pacing produced significantly more elevated diastolic Ca^2+^ levels in CPVT CMs.

In CPVT CMs significantly lower SR Ca^2+^ load was seen at baseline and in the presence of pacing during adrenaline (Figure 4C). Caffeine-induced Ca^2+^ release via RyR2 was studied under the four different aforementioned conditions (Figure 4A). To determine the fractional Ca^2+^ release, the amplitude of the Ca^2+^ transients were divided by the amplitude of the following caffeine-induced Ca^2+^ transient. Fractional SR Ca^2+^ release was significantly higher in CPVT CMs during spontaneous beating and during electrical pacing with and without adrenaline perfusion (Figure 4D).

### Current-clamp Reveals DADs and EADs in *CPVT-P2328S* CMs

Using the perforated patch technique in current-clamp mode APs of 16/18 control and 14/14 CPVT CMs were ventricular-like. The basic AP characteristics were similar in control and CPVT CMs (Table 2). Eleven CPVT and five control CMs were exposed to adrenaline and a similar increase in beating rate and decrease in APD_50_ and APD_90_ were observed in all of them. In general, control CMs had robust synchronized beating throughout the recordings, but in 3/16 CMs (19%) DADs were randomly observed during baseline recordings (1–2 DADs/60 APs). At baseline DADs were observed in 6/14 (42%) of CPVT CMs. In 6/11 CPVT CMs exposed to adrenaline, wash-out recovered the beating rate to normal as in control CMs. However, in the remaining 5/11 CPVT CMs, adrenaline subsequently evoked DADs and resulted in decreased beating rate (Figure 5A and 5B).

In three other CPVT CMs spontaneous EADs were seen at baseline (Figure 5C and 5D). Additionally phase 3 burst episodes were seen in one cell showing EADs and DADs (Figure 5C). All solitary EAD upstrokes were seen above −25 mV. The MDP of the burst was −50 mV. The maximum upstroke amplitude for solitary EADs was 45 mV and during the bursts 95 mV. No EADs or spontaneous bursting were observed in control CMs.

### MAP Recordings of *CPVT-P2328S* Patients Reveal EADs and DADs, ECGs Show Simultaneous T2 and U-waves

We examined MAP recordings for EADs and DADs and 24h-ECGs for their ECG counterparts, T2 and U-waves. MAP recordings demonstrated DADs (Figure 6A) and EADs (Figure 6B). 24h-ECGs showed occasional simultaneous T1, T2, and U-waves (Figure 6C). These changes were observed repeatedly, and no similar changes were seen in healthy controls.

## Discussion

We report, for the first time, that in addition to DADs, CPVT patient-specific iPSC-derived CMs display EADs, providing novel insight into the arrhythmogenic mechanisms in CPVT. Our findings demonstrate the applicability of iPSC-derived CMs in studying the pathophysiology of CPVT-causing *RyR2* mutations.

CPVT CMs show disturbances in intracellular Ca^2+^ cycling in response to catecholaminergic stimulation with adrenaline. These changes in Ca^2+^ cycling indicate increased diastolic SR Ca^2+^ leak, which may lead to DADs and the generation of triggered arrhythmias [13]. In accordance with a previous report [14], upon perfusion with adrenaline, CPVT CMs develop frequent DADs, which occasionally suppress the following AP, preventing the increase in the beating frequency.

Adrenaline produced significantly more elevated diastolic Ca^2+^ levels in CPVT CMs. Adrenaline also failed to increase caffeine-induced Ca^2+^ release and fractional Ca^2+^ release compared to control cells. The Ca^2+^ measurements with adrenaline were recorded after 3 minutes of perfusion with the drug. During this time Ca^2+^ leaked from the SR to the cytosol in the CPVT CMs, as indicated by the elevated diastolic Ca^2+^ levels in the cytosol and the reduced caffeine-induced SR Ca^2+^ release.

Fractional SR Ca^2+^ release increases steeply with elevation of SR calcium. [15], [16]. Therefore, it is expected that continuous adrenaline perfusion without pacing will only transiently increase fractional SR Ca^2+^ release in the RyR2 mutant CMs, which will soon find a new equilibrium, balanced by increased sensitivity to SR Ca^2+^ and decreased SR Ca^2+^ stores. On the other hand, transient pacing (10 beats) increases SR Ca^2+^ load. When the fractional SR Ca^2+^ release is measured immediately after the pacing, increased values are observed. As expected, in this case the fractional SR Ca^2+^ release is greater in the RyR2 mutant CMs, which are more sensitive to luminal SR Ca^2+^ release.

Traditionally, EADs have been thought to result from spontaneous reactivation of the L-type Ca^2+^ channel (LTCC) during conditions of prolonged APD, such as LQT2 [17]. However, recent understanding highlights the role of Ca^2+^ overload and spontaneous Ca^2+^ release as the main triggers behind EADs [18], [19]. Conditions leading to Ca^2+^ overload include heart failure, digitalis toxicity, and CPVT. Under conditions of SR Ca^2+^ overload or leaky RyR2s, spontaneous release of Ca^2+^ from the SR leads to activation of the sodium-calcium-exchanger (NCX), which results in a depolarizing current that reactivates the LTCC, leading to an EAD. It has been shown that early Ca^2+^ aftertransients are the primary events that induce EADs, and not vice versa [20], [21]. In addition to early EADs, late EADs arising from membrane potentials more negative than the threshold potential of LTCC (−35 mV), are reported to be NCX-mediated and share similar properties with DADs [22]–[24]. Further support for the role of cytosolic calcium in EAD induction comes from recent findings that despite prolonging APD, ranolazine suppresses EADs by stabilizing RyR2 [25]. However, ranolazine’s ability to prevent EADs is also likely to be mediated by late sodium current inhibition, which decreases cytosolic Ca^2+^ by reducing NCX-mediated Ca^2+^ influx [26], [27]. Lower cytosolic Ca^2+^ will less likely cause SR Ca^2+^ leak, which would lead to forward-mode NCX activation and afterdepolarizations. Accordingly, we must acknowledge the potential role of the late sodium current in EAD provocation.

Our results support this emerging consensus on the role of NCX-mediated generation of EADs. We found that CPVT CMs display irregular spontaneous calcium release events, DADs, and EADs. Furthermore, MAP recordings in *CPVT-P2328S* patients show both DADs and EADs. Although not experimentally shown, it has previously been suggested that CPVT patients with *RyR2* mutations are susceptible to both EAD and DAD-mediated arrhythmia mechanisms [10]. As shown here, these patients show both T2 and U-waves, the ECG equivalents of EADs and DADs, respectively [10].

We could not demonstrate increased arrhythmogenicity with pacing. At baseline, pacing stabilized beating in both control and CPVT CMs. When arrhythmias were provoked in CPVT CMs with adrenaline, pacing on top of that did not have any affect on the recorded arrhythmias. This is contrary to previous CPVT reports using either spontaneously beating CMs with a CASQ2 mutation [14] or resting CMs with a *RyR2* mutation [4]. This observation suggests that there are mutation-specific differences. Our *P2328S-RyR2* mutation presents arrhythmias only in the presence of catecholaminergic stimulation, but not if increased beating rate is generated by pacing.

Our findings demonstrate that in addition to DADs, *CPVT-P2328S* CMs display EADs which may be involved in arrhythmogenesis in these patients. This broadens the mechanistic understanding of arrhythmias linked to *RyR2* mutations and helps to direct efforts to optimize therapy in these patients. Our iPSC-derived CM model offers a promising platform for further research into the pathophysiological mechanisms of CPVT, as well as a safe tool for screening and optimizing drug therapy with patient-specific CMs.

### Study Limitations

We studied iPSC-derived CMs from two CPVT and two control iPS cell lines. However, both CPVT lines were from the same patient. The control lines were from two healthy controls. It is therefore unclear whether the changes we saw are typical of all CPVT patients, of this specific mutation, or only this specific patient. In the future we need to extend our studies, looking at various RyR2 mutations and several cell lines from multiple patients harboring a specific mutation.

The type of CM (nodal, atrial, or ventricular) under investigation was unclear in the Ca^2+^ imaging studies. Part of the variability in the results may stem from differences between the CM types. To address this in the future, simultaneous recording of APs and intracellular Ca^2+^ will help to distinguish cell type and additionally give temporally synchronized info on the interplay between Ca^2+^ handling and APs.

Additionally, further development of cardiac differentiation and maturation procedures will hopefully improve the homogeneity of iPS cell lines.
